# Root Microbiota in Primary and Secondary Apical Periodontitis

**DOI:** 10.3389/fmicb.2018.02374

**Published:** 2018-10-09

**Authors:** Serge Bouillaguet, Daniel Manoil, Myriam Girard, Justine Louis, Nadia Gaïa, Stefano Leo, Jacques Schrenzel, Vladimir Lazarevic

**Affiliations:** ^1^Endodontics Unit, Section of Dental Medicine, Faculty of Medicine, University of Geneva, Geneva, Switzerland; ^2^Faculty of Medicine, Genomic Research Laboratory, Service of Infectious Diseases, Geneva University Hospitals, Geneva, Switzerland

**Keywords:** apical periodontitis, endodontics, *Enterococcus faecalis*, *Fusobacterium nucleatum*, 16S rRNA gene, community profiling, oral microbiome

## Abstract

Apical periodontitis is an inflammatory disease of the dental periradicular tissues triggered by bacteria colonizing necrotic root canals. Primary apical periodontitis results from the microbial colonization of necrotic pulp tissues. Secondary apical periodontitis results from a persistent infection of incorrectly treated root canals. The aim of this study was to characterize the microbiota present in primary and secondary intraradicular infections associated with apical periodontitis using 16S rRNA gene amplicon sequencing. Teeth exhibiting apical periodontitis with or without root canal treatment were extracted after informed consent. From each tooth, the intraradicular content as well as a dentin sample (control) were collected and subjected to DNA extraction. PCR amplicons of the V3–V4 region of the bacterial 16S rRNA gene were pooled and sequenced (2 × 300) on an Illumina MiSeq instrument. The bioinformatics analysis pipeline included quality filtering, merging of forward and reverse reads, clustering of reads into operational taxonomic units (OTUs), removal of putative contaminant OTUs and assigning taxonomy. The most prevalent and abundant OTU in both dentin and root canal samples was assigned to anaerobic bacterium *Fusobacterium nucleatum*. Multivariate analysis showed clustering of microbiota by sample type (dentin vs. intraradicular content) and, in root canals, by pathology (primary vs. secondary infection). The proportions of *Enterococcus faecalis* and *F. nucleatum* were, respectively, higher and lower when comparing secondary to primary infected root canals. Co-occurrence network analysis provided evidence of microbial interactions specific to the infection type. The identification of bacterial taxa differentially abundant in primary and secondary intraradicular infections may provide the basis for targeted therapeutic approaches aimed at reducing the incidence of apical periodontitis.

## Introduction

Apical periodontitis is an inflammatory disease of dental periradicular tissues triggered by oral pathogens invading necrotic root canals. The role of bacteria in the etiology of apical periodontitis was first demonstrated by Kakehashi et al. (1965) who used germ-free and conventional laboratory rats to compare the inflammatory reactions in surgically exposed dental pulps. Whereas no apical periodontitis was detected in germ-free rats, all conventional laboratory rats developed a pulpal necrosis associated with a severe inflammatory reaction around periapical tissues. Results from Sundqvist (1992) further supported the concept of a selective environment in necrotic root canals that modulates the synergistic and antagonistic activities of colonizing pathogens. Although classic studies showed that approximately 200–300 bacterial species can be cultured from samples collected in the oral cavity, only few of these species have been isolated from necrotic root canals. Strictly anaerobic bacteria were shown to dominate the microbial population of untreated necrotic root canals, with approximately 5–12 genera including *Peptostreptococcus, Prevotella, Porphyromonas, Fusobacterium, Eubacterium*, and *Actinomyces* along with facultative anaerobic streptococci. The presence of such pathogens in necrotic root canals was shown to fairly correlate with the observation of apical inflammatory lesions on intraoral radiographs (Bergenholtz, 1974; Fabricious et al., 1982; Ando and Hoshino, 1990; Chávez de Paz, 2005). This so-called “primary apical periodontitis” generally heals upon completion of a root canal treatment that combines a chemo-mechanical debridement of infected tissues with a root canal filling. Unsuccessfully treated root canals may exhibit a persistent inflammation called “secondary apical periodontitis.” Treated roots associated with secondary apical periodontitis have microbial populations distinct from those of untreated roots and culture-based studies have rarely identified more than 1–3 genera of Gram-positive facultative anaerobes, including *Streptococcus, Lactobacillus*, and *Enterococcus* (Sundqvist et al., 1998). Some of these bacteria were shown to resist the action of conventional antimicrobial agents and to survive in root-filled teeth for many years. A considerable interest has been focused on *Enterococcus faecalis*, a bacterium frequently isolated as a monoculture in root-filled teeth but rarely identified in untreated root canals (Stuart et al., 2006; Chávez de Paz et al., 2007). Clinical studies reported that 30–65% of root-filled teeth may show a radiographic evidence of secondary apical periodontitis and that apical periodontitis may exacerbate several systemic diseases (Khalighinejad et al., 2016).

For many years, knowledge of bacteria colonizing different ecological niches of the oral cavity was limited to those species that could be cultured in the laboratory. As an example, only 40–50% of the bacteria present in the human subgingival plaque can be cultured *in vitro* (Paster et al., 2001). A similar observation was made by Anderson et al. (2012) who combined culture-dependent and -independent approaches to study the microbiota in root-filled teeth associated with periradicular lesions. The authors showed that the two methods used yielded different results and highlighted the benefit of open-ended molecular methods for the assessment of microbial diversity in apical periodontitis. They also pointed out the risk of underestimating the role of as-yet-uncultivated species in the etiology of apical periodontitis.

Latest molecular methods that rely on high-throughput sequencing of the 16S ribosomal RNA (rRNA) gene are now increasingly used to provide a more comprehensive overview of the microbiota from different sites of the human body including the oral cavity. This approach identified taxa not yet discovered in oral microbiota samples (Keijser et al., 2008; Lazarevic et al., 2009) and showed that the bacterial diversity in the oral cavity is larger than thought before (Siqueira and Rôças, 2017). Rôças et al. (2016) used the same methodology to analyze the microbiota of deep dentinal caries in symptomatic teeth.

The aim of this study was to characterize the microbiota present in primary and secondary intraradicular infections associated with apical periodontitis using 16S rRNA gene amplicon sequencing. A deeper knowledge of these microbiota would contribute to the development of more effective therapeutic procedures and help to reduce the high incidence of non-healing necrotic teeth.

## Materials and Methods

### Case Selection

The microbiological samples were collected from teeth scheduled for extraction at the Department of Oral Surgery of the Geneva University Hospitals. Only teeth presenting with periapical lesions visible on intraoral radiographs were included in this study (PAI score ≥ 3, Orstavik et al., 1986). Teeth associated with advanced periodontal lesions or extensive crown destruction allowing permanent contamination from the oral cavity, were excluded from the analysis. Patients with extremely poor oral hygiene, severe general health status or patients who received antibiotics within 2 months preceding the extraction of the tooth were not included in the study. Teeth were divided in two groups; either teeth exhibiting necrotic pulps without prior root canal treatment (primary apical periodontitis group – PAP) or teeth displaying a pre-existing root canal treatment (secondary apical periodontitis group – SAP). This study protocol was approved by the Ethical Committee for human research of the state of Geneva (number 14–199), and all patients gave their written informed consent.

### Samples Collection and Processing

Prior to tooth extraction, all patients were given a 0.2% chlorhexidine mouth rinse for 1 min and care was taken to perform surgical procedures under aseptic conditions. Immediately after extraction, the tooth was placed in a sterile compress and transported to the laboratory. The external surface of the root was gently cleansed for 20 s using an ultrasonic dental scaling tip (Piezon Master 700, E.M.S. Electro Medical Systems S.A., Nyon, Switzerland) under molecular grade water irrigation (Sigma-Aldrich Chemie GmbH, Buchs, Switzerland). The apical 5 mm of the root were then cut with a sterile diamond-coated disk (NTI Serrated Diamond Discs, Kerr Dental). Only this portion of the root was used for the intraradicular bacterial sampling. The intraradicular content was harvested from the canal under a laminar hood using a #15 dental file (K-files, Micro-Mega) and collected into a 1.5 mL tube (DNase/RNase free, Axygen Biosciences). The file used to collect the intraradicular content was placed in the same tube. For each tooth, external dentin chips and an unused sterile file were collected into separate tubes and used as controls.

### DNA Extraction

DNA was extracted using Extract-N-Amp Plant PCR Kit (Sigma-Aldrich) which may prove useful for samples with low biomass, since this approach does not include DNA clean-up associated with high DNA loss (Videvall et al., 2017). The root canal material (together with the file used for its collection) and dentin chips obtained from each extracted tooth were added to 100 μL of Extract-N-Amp Plant Extraction solution and heated at 95°C for 10 min. One hundred micro liter of Extract-N-Amp Plant Dilution solution were added and mixed by vortexing. To control for the reagent DNA contamination, an unused file (negative control) was processed in parallel with dentin and root canal samples for each tooth. Crude DNA extracts were kept at –20°C until further processing.

### Amplicon Sequencing

The V3–4 region of the bacterial 16S rRNA genes (*Escherichia coli* positions 341–805) was amplified using 5 μL of DNA extract obtained from clinical (dentin and root canal) samples and negative controls in a 20 μl volume of KAPA2G Robust HotStart ReadyMix (Kapa Biosystems) containing each of 0.4 μM forward primer 341F 5′-CCTACGGGNGGCWGCAG-3′ and reverse primer 805R 5′-GACTACHVGGGTATCTAAKCC-3′. The PCRs were carried out with an initial denaturation at 95°C for 3 min, followed by 33 cycles of denaturation at 95°C for 30 s, annealing at 51°C for 30 s, and extension at 72°C for 60 s, and a final extension at 72°C for 5 min. Duplicate PCRs of each sample were combined and run (1 μL) on a 2100 Bioanalyzer (Agilent Technologies, Santa Clara, CA, United States) for quality analysis and quantification. The amplicon barcoding/purification and construction of the sequencing library were performed as described previously (Lazarevic et al., 2016). Paired-end sequencing was carried out for 300 cycles on an Illumina MiSeq instrument using MiSeq v3 Reagent Kit at LGC Genomics (Berlin, Germany).

### Sequence Analysis

Reads with incorrect barcodes, missing barcodes, or conflicting barcode pairs were discarded. After removal of adapter remnants and primer sequences using proprietary LGC Genomics software, sequencing data were submitted to the European Nucleotide Archive ^1^ under the project number PRJEB26080. Paired reads were quality filtered and joined using PEAR v. 0.9.10 (-m 460 -n 390 -t 240 -v 20 -q 33 -p 0.0001 -u 0) (Zhang et al., 2014). Merged sequence reads were clustered into operational taxonomic units (OTUs) at the 97% similarity threshold using the UPARSE pipeline (Edgar, 2013) implemented in USEARCH v. 8.1.1861 (Edgar, 2010). This pipeline removes singleton reads before creating OTUs and filter out putative chimeric sequences.

We removed from the sample dataset OTUs that matched any of the following criteria: (i) had higher average relative abundance in negative extraction controls than in root canal samples; (ii) remained unclassified at the phylum level; (iii) presented <90% identity to reference EzBioCloud 16S database sequences as revealed by USEARCH (-usearch_local -id 0.9 -query_cov 0.99). The dataset was normalized to the same number of reads across all samples using the ‘rrarefy’ command of the R vegan package. A representative sequence of each OTU was classified using EzBioCloud 16S database (Yoon et al., 2017) via mothur’s (Schloss et al., 2009) command classify.seq (-method wang -cutoff 80). For control and comparison purposes, each representative OTU sequence was also compared against the EzBioCloud 16S database (downloaded on September 5, 2017) and Human Oral Microbiome Database (HOMD) 15.1 (Chen et al., 2010) using USEARCH (-usearch_global -id 0.9 -evalue 0.00001 -top_hits_only) and the taxonomy of the top hit reference sequence(s) was reported.

For prediction of functional profiles of bacterial communities, representative sequences of OTUs from the decontaminated and normalized data set were mapped to the Greengenes 16S rRNA gene reference database (13.5) (McDonald et al., 2012) pre-clustered at 97% identity using USEARCH (-usearch_local -id 0.97 -query_cov 0.9 -top_hit_only). Phylogenetic Investigation of Community by Reconstruction of Unobserved States (PICRUSt) v.1.1.3 (Langille et al., 2013) was used to normalize the data by 16S rRNA gene copy numbers and derive Kyoto Encyclopedia of Genes and Genomes (KEGG) Orthology (Ogata et al., 1999) abundance data. Functional profiles were compared with STatistical Analysis of Metagenomic Profiles (STAMP) v2.1.3 (Parks et al., 2014).

### Comparison of Bacterial Communities

Microbiota comparisons were carried out using Bray–Curtis similarity (Bray and Curtis, 1957). The similarity matrix, based on the square-root-transformed relative abundance of OTUs was constructed in PRIMER (Primer-E Ltd., Plymouth, United Kingdom). Principal coordinates analysis (PCoA) and group average hierarchical clustering of Bray–Curtis similarity matrices were performed in PRIMER. Shannon diversity index (H’ log_e_) was calculated from the relative abundance of OTUs in PRIMER.

### Statistical Analysis

To assess differences between different sample types (dentin, root canal), pathologies (PAP, SAP) we used PERmutational Multivariate ANalysis Of VAriance (PERMANOVA, PRIMER) of the Bray–Curtis similarity matrix. Canonical Analysis of Principal Coordinates (CAP, PRIMER) (Anderson and Willis, 2003), based on the Bray–Curtis similarity matrix was used to test whether the pathology (PAP and SAP) could be predicted from the microbiota profiles. PERMANOVA and CAP were run with 9,999 permutations. Wilcoxon rank-sum test was used to assess statistical significance of differences in the relative abundance of individual taxa. Statistical significance was set at the 95% confidence level (*P* < 0.05). Co-presence and mutual exclusion of bacterial taxa were assessed by CoNet (Faust et al., 2012) using Cytoscape plugin (Shannon et al., 2003). The Spearman’s rank correlation coefficient values >0.5 and <-0.5 were considered to reflect significant positive and negative correlations.

## Results

### Sequencing of 16S rRNA Gene Amplicons

Illumina sequencing of the 16S rRNA V3–4 amplicon libraries generated from 52 root canals, 52 dentin and 52 negative controls yielded 6,950,125 raw read pairs of which 6,058,205 were joined and passed the quality control steps. After removal of the reads from contaminant OTUs (**Supplementary Table S1**), whose proportions were significantly higher in dentin than in root canal samples (45 vs. 14.6%), we normalized the sequence data set to 14,000 reads per sample. We discarded samples that did not reach this threshold, which was chosen based on the distribution of the number of sequences obtained per root canal samples and the trade-off between sequencing depth and sample size of PAP and SAP groups. The remaining 43 dental roots (of which 21 were associated with PAP and 22 with SAP) and 21 dentin samples were further analyzed. They contained 347 and 303 OTUs, respectively, of which 276 were shared by the two sample types (**Supplementary Table S2**).

### Taxonomic Composition of Root Canal and Dentin Samples

**FIGURE 1:**
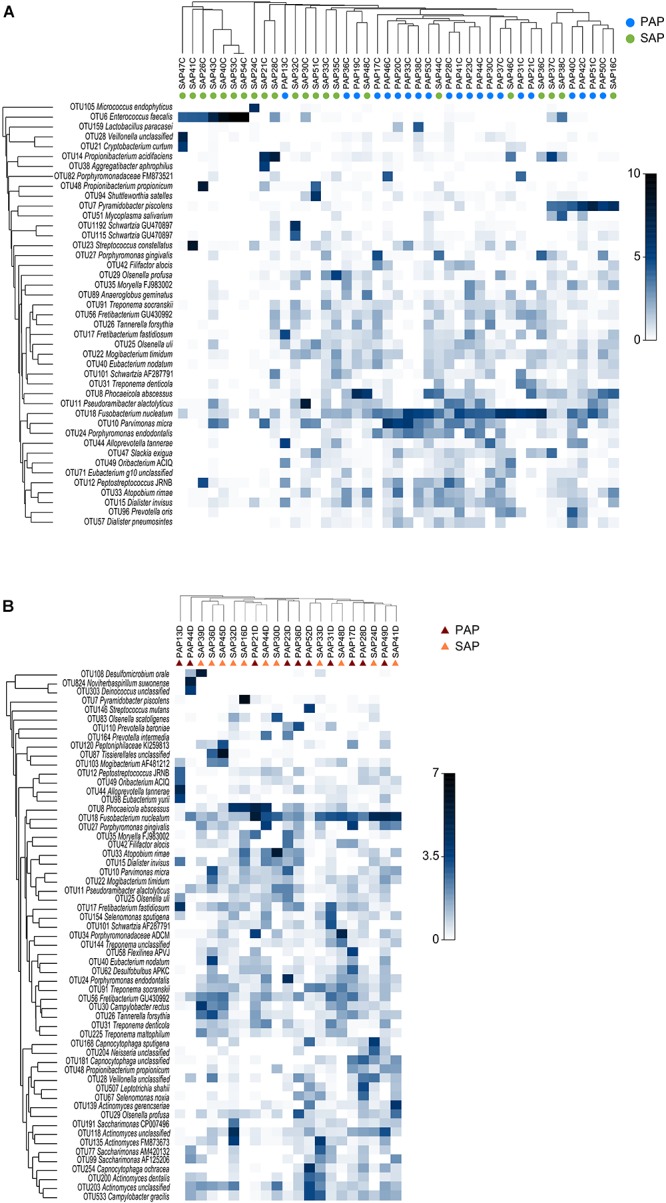
Taxonomic composition and group average clustering of bacterial communities in root canal **(A)** and dentin **(B)**. The heat map shows the relative abundance of OTUs across samples. OTUs with average relative abundance >0.5% in a given sample type are presented. The hierarchical clustering was based on square root-transformed proportions of OTUs and Bray–Curtis similarity matrix. PAP, primary apical periodontitis; SAP, secondary apical periodontitis.

**FIGURE 2:**
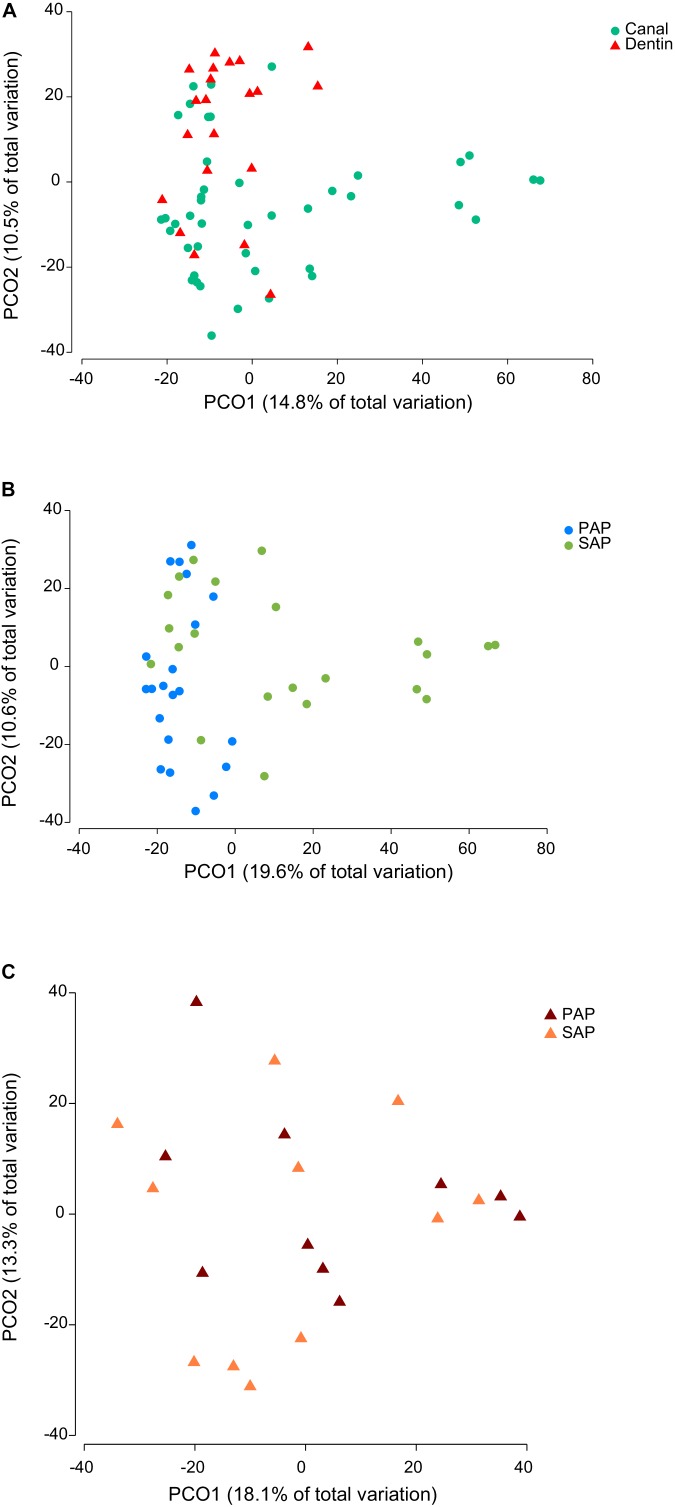
Principal coordinate analysis of Bray–Curtis similarity of bacterial communities. The analysis was based on square root-transformed proportions of OTUs and included either all **(A)**, root canal **(B)**, or dentin samples **(C)**. PAP, primary apical periodontitis; SAP, secondary apical periodontitis.

Representative OTUs were classified against the manually curated EzBioCloud database, containing 61,933 16S rRNA gene sequences from prokaryotic species and phylotypes. The 374 OTUs identified were assigned to 18 phyla (**Supplementary Tables S2, S3**) and 177 genera (**Supplementary Tables S2, S4**). Of 16 bacterial phyla identified in root canal samples, five (Firmicutes, Bacteroidetes, Actinobacteria, Fusobacteria, and Synergistetes) corresponded together to more than 90% of the sequence reads. *Fusobacterium nucleatum* OTU18 was by far the most proportionally abundant OTU, both in dentin and root canal samples (**Figure 1**). *Phocaeicola abscessus* OTU8 was among the top five most abundant OTUs at both anatomical sites. No other OTUs were common to top ten most abundant OTUs in dentin and root canals. We also compared the prevalence (i.e., the presence regardless of the relative abundance) of OTUs in two sample types. Again, *F. nucleatum* OTU18 was most prevalent, being detected in all but one (root canal) sample. Three other OTUs, *Mogibacterium timidum* OTU22, *Pseudoramibacter alactolyticus* OTU11 and *Fretibacterium*_GU430992 OTU56 were among ten most prevalent OTUs in both sample types. However, overall microbiota profiles suggested that dentin and canal samples had distinct microbiota. Principal coordinate analysis (PCoA) of Bray–Curtis similarity showed microbiota clustering by sample type (**Figure 2A**), where dentin and root canal samples are mainly associated with positive and negative PCo-2 values, respectively. PERMANOVA analysis confirmed statistically significant (*P* = 0.0003) differences in microbiota profiles from the two sample types. However, inter-site (dentin vs. root canal) microbiota Bray–Curtis similarities varied among the subjects from 5.5 to 90 (on a 0–100 scale) with an average value of 50.5 (**Supplementary Table S5**). The three individuals with highest (>80) inter-site Bray–Curtis similarity (PAP13, SAP16, and SAP44) did not show much similarity between each other in terms of bacterial community composition (**Figure 1**).

### Microbiota Profiles in Relation to Primary and Secondary Infections

To assess similarities and differences between microbiota associated with PAP and SAP, we performed PCoA separately for each sample type. No obvious clustering by pathology was observed for dentin samples (**Figure 2C**), which was in accordance with the result of the PERMANOVA test (*P* = 0.8478). Conversely, PAP and SAP cases of root canals were relatively well separated in first two PCo (**Figure 2B**) and differences in microbiota profiles between the two pathologies were significant (PERMANOVA *P* < 0.0001). Canonical analysis of principal coordinates, a method that maximizes differences between pre-defined groups (PAP and SAP) showed that the two pathologies may be well predicted from the root canal microbiota OTU profile, with 90.5 and 77.2% correct allocations for PAP and SAP, respectively (*m* = 9, δ_1_ = 0.7894, *P* = 0.0002).

Hierarchical cluster analysis or root canal samples revealed two major clusters. One of them consisted of seven SAP samples (**Figure 1A**) with low bacterial diversity, containing relatively high levels (17–99.9%) of *E. faecalis* OTU6. The second major cluster contained 36 samples generally dominated by bacteria other than *E. faecalis*, notably *Pyramidobacter piscolens* OTU7 and *F. nucleatum* OTU18. A sub-group of this second major cluster was mainly formed of PAP samples (10/11) with *E. faecalis* OTU6 being undetected or found at low relative abundance (<0.04%).

**FIGURE 3:**
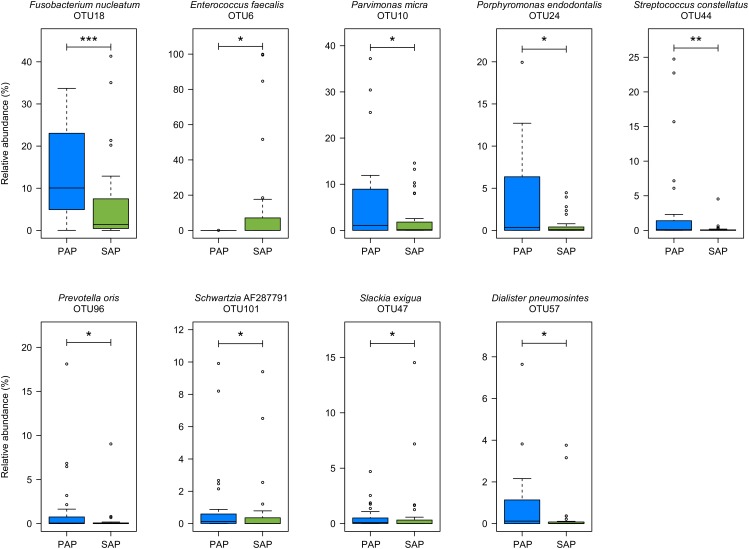
OTUs differentially represented between PAP and SAP root samples. OTUs with significant changes (Wilcoxon rank-sum test, *P* < 0.05) and a minimum average relative abundance ≥0.5%) are presented. Boxplots show the first and third quartile (top and bottom edges of the rectangle) divided by median. Whiskers correspond to the highest and lowest values within 1.5× the interquartile range. Outliers are shown as circles. ^∗^0.01 < *P* < 0.05; ^∗∗^0.001 < *P* < 0.01; ^∗∗∗^*P* < 0.001. PAP, primary apical periodontitis; SAP, secondary apical periodontitis.

Several OTUs were differentially abundant between the two pathologies (**Figure 3**) as revealed by Wilcoxon rank-sum test. The relative abundances of OTUs assigned to *F. nucleatum* (OTU18), *Parvimonas micra* (OTU10), *Porphyromonas endodontalis* (OTU24), *Prevotella oris* (OTU96), *Slackia exigua* (OTU47), *Dialister pneumosintes* (OTU57), and *Schwartzia*_AF287791 (OTU101), which are obligate anaerobes, and to *Streptococcus constellatus* (OTU44) which may be anaerobic or capnophilic, were higher in PAP as compared to SAP samples. In contrast, the proportion of OTU6 assigned to *E. faecalis*, a facultative anaerobe, was higher in SAP than in PAP samples.

Community differences between the two pathologies were also observed at the phylum level. Of 16 bacterial phyla identified in root canal samples, three (Bacteroidetes, Fusobacteria, and Spirochaetes) had significantly higher relative abundance in PAP as compared to SAP samples (**Supplementary Table S3**). Phylum Actinobacteria was significantly enriched in SAP.

**FIGURE 4:**
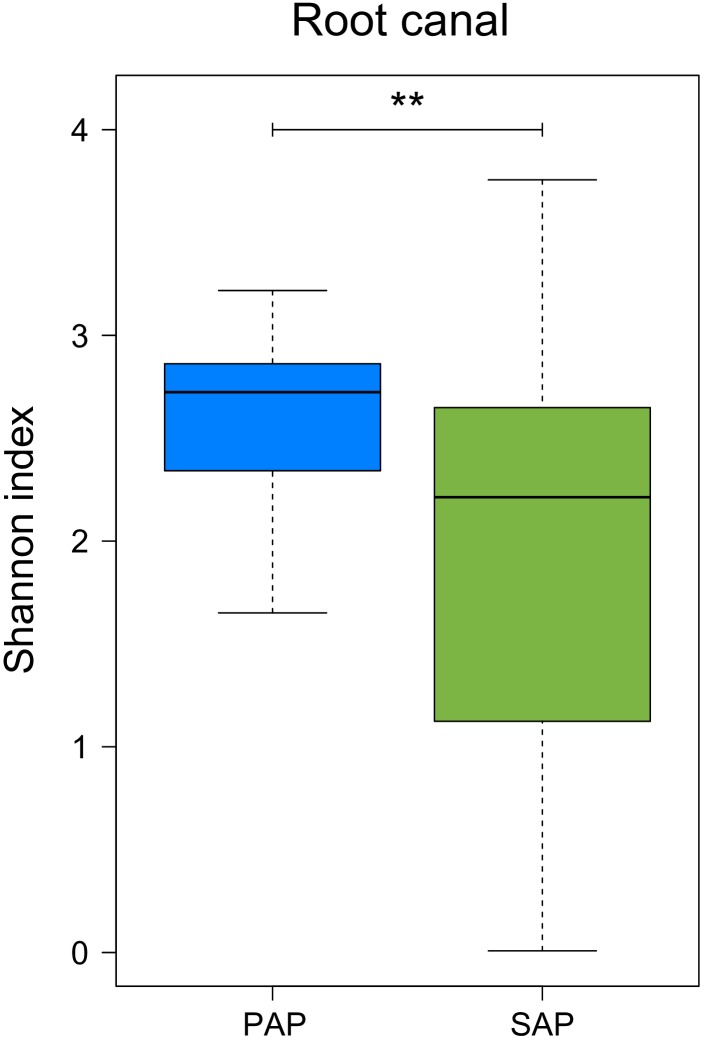
Alpha diversity of the microbiota from root canal of PAP and SAP teeth. Shannon diversity index was based on OTU relative abundance. ^∗∗^0.001 < *P* < 0.01. PAP, primary apical periodontitis; SAP, secondary apical periodontitis.

Our data also showed that bacterial diversity in root samples, measured as Shannon diversity index, was significantly higher (Wilcoxon rank-sum test, *P* = 0.0094) in PAP as compared to SAP (**Figure 4**).

### Patterns of Microbial Communities

**FIGURE 5:**
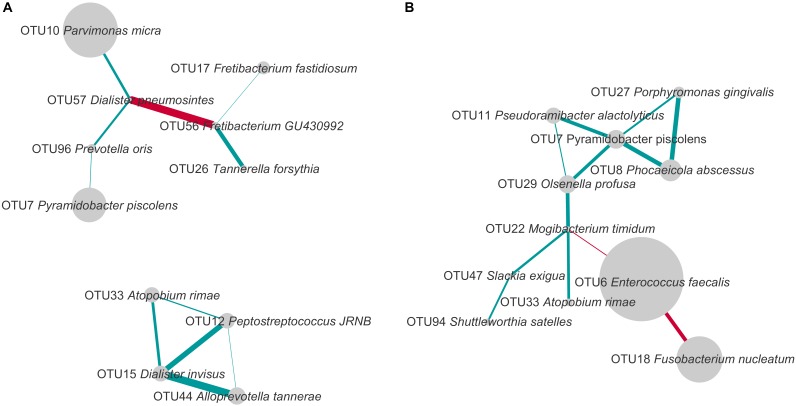
Co-occurrence and exclusion patterns among most abundant OTUs in PAP **(A)** and SAP **(B)** in root canal samples. OTUs found in at least eight samples and with a minimal average relative abundance of 1% (considering all root canal samples) were analyzed. The edges represent positive (blue) and negative (red) Spearman correlations. Only correlations with Spearman *R* > 0.5 or *R* < –0.5 are presented. The line thickness is proportional to the absolute value of the Spearman’s correlation. Node sizes reflect average relative abundance of each OTU. PAP, primary apical periodontitis; SAP, secondary apical periodontitis.

To study the co-occurrence and exclusion of OTUs, we performed a network analysis separately for PAP and SAP root canal samples. Our results revealed both positive and negative associations between OTUs (**Figure 5**).

In PAP samples, *D. pneumosintes* OTU57 negatively correlated with *Fretibacterium*_GU430992 OTU56, but both of them displayed positive associations with other OTUs. In the second disconnected sub-network, another OTU of genus *Dialister*, *D. invisus* OTU15, positively correlated with three OTUs.

In secondary infected root canals, *E. faecalis* OTU6 negatively correlated with both *F. nucleatum* OTU18 and *Mogibacterium timidum* OTU22. In addition, 10 positive correlations including nine OTUs were identified. *P. piscolens* OTU7 showed co-occurrence with four OTUs and was central in a sub-network of five OTUs that belong to four different phyla and present multiple positive correlations.

The only organism present in correlation networks (as defined using cut-offs described in **Figure 5**) of both PAP and SAP was *P. piscolens* OTU7. Its associations with other community members differed between the two pathologies.

### Prediction of Functional Profiles of Bacterial Communities

**FIGURE 6:**
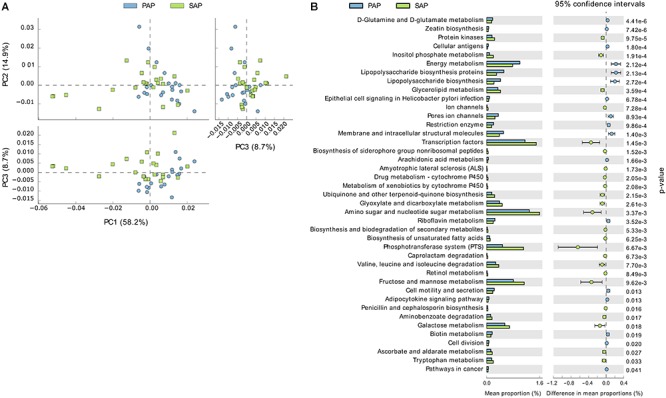
Functional profiles of PAP and SAP-associated bacterial communities in root canal samples. The KEGG orthologs (predicted functions) inferred from the relative abundance of OTUs using PICRUSt were collapsed into level-3 KEGG ontology (KEGG pathways) and compared among PAP and SAP groups using STAMP. **(A)** Principal component analysis of KEGG pathways. **(B)** Significant differences (*P* < 0.05, two-sided Welch *t*-test) between PAP and SAP functions. STAMP was set to consider pathways represented by at least 10 sequences and an effect size filter (ratio of proportion) of 1.2. PAP, primary apical periodontitis; SAP, secondary apical periodontitis.

PAP and SAP samples were relatively separated in principal component analysis based on the PICRUSt data (**Figure 6**). This analysis showed that several KEGG pathways differed between the two groups. Notably, gene families involved in phosphotransferase systems and the metabolism of galactose, fructose/mannose, amino sugars, nucleotide sugars and glycerolipids were enriched in SAP, while those responsible for lipopolysaccharides biosynthesis were significantly associated with PAP.

## Discussion

The results of the current study, performed on 43 dental roots, support the polymicrobial etiology of apical periodontitis and confirm that distinct bacterial communities are found in PAP and SAP.

The composition of bacterial communities identified in the current study was globally similar to those found in previous studies of PAP and SAP by 16S rRNA amplicon sequencing (Hong et al., 2013; Tzanetakis et al., 2015; Keskin et al., 2017). However, the proportion of certain bacterial taxa, the presence of taxa (phyla and genera) discriminating between PAP and SAP, and differences in bacterial diversity between these pathologies varied among studies. For example, in one study (Keskin et al., 2017), Proteobacteria was a dominant phylum whereas in others (Hong et al., 2013; Tzanetakis et al., 2015), including ours, it was much less represented. In agreement with the Tzanetakis study (Tzanetakis et al., 2015), we found that root canal microbiota associated with SAP harbored higher levels of Proteobacteria and Tenericutes than those from PAP patients (**Supplementary Table S3**). However, these differences did not reach the threshold of statistical significance, whereas other phyla showed significant shifts related to the type of infection. In line with Keskin study (Keskin et al., 2017), SAP was associated with significantly higher proportion of *Enterococcus* and lower proportions of *Prevotella* and *Slackia* (**Supplementary Table S4**). A significant decrease in bacterial diversity in our SAP samples compared with PAP was not common to other studies which reported an increased diversity of SAP (Tzanetakis et al., 2015) or no significant difference between the two types of infection (Hong et al., 2013; Keskin et al., 2017).

The variations between root canal microbiota across studies may be due to a combination of factors including differences in inclusion/exclusion criteria, tooth treatments or methodologies used to sample the microbiota. For example, only the bacterial content of the apical portion of the root was used in this study, owing to the fact that bacteria in close vicinity of the periapical tissues were more likely to elicit apical periodontitis (Özok et al., 2012). Also, care was taken to control for contamination of the tooth samples with saliva during tooth extraction. The differences in taxonomic composition of bacterial communities found in many root canal–dentin pairs of samples show that dentin microbiota cannot be used as a proxy for root canal microbial communities and suggest that no external contamination of root canals has occurred during sampling (**Figure 1**). This control is not possible when samples are prepared for cryopulverization and crushed in a cryogenic grinder to collect dentin powder for analysis (Siqueira et al., 2011; Keskin et al., 2017). In addition, DNA extraction methods, strategies used to amplify the 16S rRNA gene and bioinformatics pipelines may also have contributed to variations among studies. Finally, all above-mentioned studies have been conducted in different countries, and therefore the influence of geographic-related factors, including environmental contaminants from food or other extraoral sources cannot be ruled out (Zehnder and Guggenheim, 2009; Anderson et al., 2015; Tzanetakis et al., 2015).

The current study further attempted to analyze the co-presence or exclusion of OTUs within the two pathologies. Several bacterial taxa present in networks of interactions in PAP or SAP have been previously associated with oral dysbiosis. For example, OTU17, found in the network of interactions in PAP, was assigned to *Fretibacterium fastidiosum* a recently described species shown to belong to the dysbiotic oral microbiome in periodontal disease and to resist conventional endodontic therapy (Rôças et al., 2014; Deng et al., 2017). *D. invisus* OTU15 and *D. pneumosintes* OTU57 that establish multiple interactions with other species in PAP (**Figure 5**), belong to the genus frequently identified as a member of the endodontic microbiota of infected root canals (Munson et al., 2002).

In SAP samples, *E. faecalis* OTU6 was the most abundant OTU detected, with proportions significantly higher than those in PAP. This may suggest that *E. faecalis*, possibly iatrogenically introduced, better resists to the environmental conditions of treated root canals. Higher functional potential of the microbiota for sugar uptake and metabolism in SAP, as compared to PAP, may also be ascribed to ecological changes due to root canal treatment.

Our network analysis of SAP samples shows a strong negative correlation between *E. faecalis* OTU6 and *F. nucleatum* OTU18 which is the most abundant species in PAP (**Figures 3, 5**). *E. faecalis* also displays negative correlation with *M. timidum* OTU22, a bacterium with higher detection frequency in the subgingival microbiota of periodontitis patients than in healthy controls (Mayanagi et al., 2004). In addition, *E. faecalis* has several mechanisms that may improve its survival in the treated root canal environment. These include its ability to form biofilms on root canal walls, to resist calcium hydroxide medication during treatment (Siqueira and de Uzeda, 1996) and to enter a viable-but-non-cultivable state (VBNC) to survive fastidious conditions (Distel et al., 2002; Evans et al., 2002; Lleò et al., 2002). In agreement with the Antunes et al. (2015) study, *E. faecalis* was not detected in all SAP samples, challenging the role of this species as a main SAP pathogen. Given the polymicrobial origin of intraradicular infections, species with central positions in the networks of interactions may be considered as possible analogs of keystone pathogens in periodontitis. Even though such organisms are not considered as “classical pathogens” causing clear-cut mono-infections, they may represent putative targets for therapeutic interventions (Rôças and Siqueira, 2012).

There is growing evidence that either PAP or SAP may exacerbate several systemic diseases including cardiovascular diseases, diabetes mellitus, chronic liver diseases and blood disorders (Caplan et al., 2006; Khalighinejad et al., 2016). A better knowledge of the microbiota involved in either PAP or SAP would help to more precisely define endodontic conditions predisposing to systemic diseases. *F. nucleatum*, the most prevalent and proportionally abundant bacterial species in PAP, is also prevalent in colorectal cancer tissues and associated distal metastases (Bullman et al., 2017). On the other hand, *E. faecalis* commonly found in many (but not all) SAP samples is one of the most common causes of nosocomial infections that can be complicated to treat because of increased antibiotic resistance, as recently confirmed by Tyson et al. (2018).

Clearly, more research is warranted to better understand associations between oral bacteria as well as their interaction with the immune system in order to delineate more efficient strategies for the elimination of endodontic pathogens and their systemic effects.

## Author Contributions

DM, MG, JL, NG, SL, and VL performed the experiments and analyzed the data. VL, SB, and JS edited the manuscript and designed, supervised, and coordinated the whole project.

## Conflict of Interest Statement

The authors declare that the research was conducted in the absence of any commercial or financial relationships that could be construed as a potential conflict of interest.

## Footnotes

1 www.ebi.ac.uk/ena
